# Corneal Remodeling After DMEK in Fuchs Endothelial Dystrophy Patients: Quantitative and Qualitative Changes

**DOI:** 10.3390/life16050805

**Published:** 2026-05-12

**Authors:** Ana Maria Arghirescu, Alina-Gabriela Gheorghe, Maria Cristina Marinescu, Dana-Margareta-Cornelia Dascalescu, Vasile Potop, Andreea-Gabriela Schmitzer, Liliana Mary Voinea, Farah Constantin, Radu Constantin Ciuluvica

**Affiliations:** 1 Doctoral School, Carol Davila University of Medicine and Pharmacy, 020021 Bucharest, Romania; 2 Department of Ophthalmology, Clinical Institute of Ophthalmological Emergencies “Prof. Dr. Mircea Olteanu”, 010464 Bucharest, Romania; 3 Department of Ophthalmology, Carol Davila University of Medicine and Pharmacy, 020021 Bucharest, Romania; 4 Medical Physiology Discipline, Carol Davila University of Medicine and Pharmacy, 020021 Bucharest, Romania; 5 Department of Ophthalmology, Faculty of Medicine, Ovidius University, 900470 Constanta, Romania; 6 Clinical Emergency Hospital “Sf Apostol Andrei”, 900591 Constanta, Romania; 7 Department of Anatomy, Faculty of Dental Medicine, Carol Davila University of Medicine and Pharmacy, 020021 Bucharest, Romania

**Keywords:** Fuchs corneal endothelial dystrophy, DMEK, anterior segment optical coherence tomography, subclinical corneal edema, corneal edema, high-order aberrations, HOAs, anterior HOAs, posterior HOAs, posterior corneal astigmatism

## Abstract

**Background/Objectives**: Fuchs endothelial corneal dystrophy (FECD) affects not only the corneal endothelium and Descemet membrane, but also the corneal thickness, shape, and optical quality. This study aimed to evaluate the quantitative and qualitative corneal changes after a Descemet membrane endothelial keratoplasty (DMEK) in patients with FECD and to compare the postoperative remodeling between eyes with subclinical corneal edema (F-SCE) and those with clinically evident corneal edema (F-CE). **Methods**: This retrospective observational study included 48 eyes of 40 patients with FECD who underwent DMEK between April 2023 and October 2025. The following parameters were assessed preoperatively and at 6 months postoperatively: central corneal thickness (CCT), mean pupillary power (MPP), anterior and posterior corneal astigmatism, total corneal higher-order aberrations (HOAs), anterior and posterior optical path difference (OPD), point spread function (PSF), anterior and posterior corneal surface regularity (RMS/A), and Gullstrand anterior/posterior (A/P) and posterior/anterior (P/A) ratios. The corneal data was obtained using a CSO MS-39 (Costruzione Strumenti Oftalmici, Firenze, Italy). **Results**: In the F-SCE subgroup, significant postoperative improvement was observed in the CCT (Δ = −84.6 ± 48.1 µm, *p* < 0.001), MPP (Δ = −1.010 ± 2.183, *p* < 0.001), posterior corneal regularity (posterior RMS/A) (Δ = −0.117 ± 0.229, *p* = 0.004), and both Gullstrand ratios (A/P: Δ = +0.111 ± 0.089, *p* < 0.001; P/A: Δ = −0.078 ± 0.064, *p* < 0.001). No significant change was detected in the anterior corneal regularity, anterior or posterior astigmatic metrics, total corneal higher-order aberrations, or PSF. In the F-CE subgroup, the postoperative response was greater, with significant improvement in the BCVA, CCT, thinnest corneal point, MPP, anterior and posterior corneal astigmatism, cylinder, total corneal HOAs, anterior and posterior corneal HOAs, PSF, anterior and posterior corneal regularity, and both Gullstrand ratios. Compared with the F-SCE eyes, the F-CE eyes showed significantly greater postoperative change in most structural and optical parameters, although most 6-month postoperative values were no longer significantly different between groups. **Conclusions**: DMEK induced significant corneal remodeling in both subclinical and clinically evident FECD. The eyes with subclinical edema demonstrated meaningful recovery in visual acuity, pachymetry, and posterior corneal regularity, supporting the presence of functional corneal impairment before a clinically visible edema. The eyes with clinically evident edema showed a broader and greater postoperative response, while the final postoperative values largely converged between groups.

## 1. Introduction

Fuchs endothelial corneal dystrophy (FECD) is a progressive, bilateral corneal dystrophy in which the corneal endothelial cells gradually degenerate, and the Descemet membrane develops characteristic guttae, leading to failure of the endothelial pump–barrier function that normally maintains corneal deturgescence [1,2]. As a result, the endothelium is the primary site of cellular loss, oxidative stress, mitochondrial dysfunction, and apoptosis-related injury, with progressive reduction in the functional endothelial cell density [3,4,5]. The resulting pump failure causes fluid accumulation within the stroma, producing corneal thickening, increased light scatter, and loss of transparency that contribute substantially to visual decline [6,7,8]. In more advanced disease, an edema extends anteriorly to involve the epithelium, causing microcystic epithelial edema and, in severe cases, painful epithelial bullae and surface irregularity. Thus, although FECD begins as a disorder of the posterior cornea, its effects ultimately involve all major corneal layers and lead to progressive visual impairment [2,9,10].

Regarding disease debut, a less frequent, early-onset subset of cases has been described, with autosomal dominant inheritance [11]. However, the predominant form of the disease is late onset (over the age of 50), also most often inherited in an autosomal dominant manner, however with variable penetrance and expressivity [12].

As most cases debut after the age of fifty, the physiological processes of corneal aging may play a concurrent role: the monolayer of endothelial cells presents with a limited division capacity, and over a normal lifespan, the cell density is expected to decrease from 5000 cells/mm^2^ at birth, to around 3000 cells/mm^2^ in the second to fourth decades of life, down to around 2600 cells/mm^2^ in the elderly, overall revealing an annual rate of 0.3–0.6% loss. The abovementioned effects of FECD are most often found under the functional threshold of 500 cells/mm^2^ [13].

The prevalence of FECD is estimated at more than 5% globally [14], making it a relatively rare disease worldwide; however, a recent meta-analysis based on four studies proposed a pooled prevalence estimate of 7.33% [15]. From this rate, the authors estimated a total of almost 300 million people worldwide over the age of 30 with FECD [15].

The prevalence of Fuchs endothelial corneal dystrophy in Romania remains unknown, and not much data is available for other European populations either: in an Icelandic cohort aged over 55 years old, the prevalence of cornea guttata was 7–11% for males and females [16], while in Czech patients undergoing cataract surgery, 29.6% were diagnosed with FECD (of which 68.8% had stage I) [17].

Fuchs endothelial corneal dystrophy (FECD) is, at the moment, the leading indication for Descemet membrane endothelial keratoplasty (DMEK) surgery [18,19], with studies showing significant post-surgical improvement in both visual acuity and visual quality [20,21]. Descemet membrane endothelial keratoplasty represents one of the most frequently performed posterior lamellar keratoplasty techniques nowadays. In addition to FECD, common indications include bullous keratopathy post cataract surgery, endothelial decompensation post glaucoma surgery and endothelial cell depletion on a penetrating keratoplasty graft [22]. Most patients achieve excellent visual recovery within the first week, with these results remaining stable even ten years post keratoplasty [23].

Currently, one of the most used classifications is based on a clinical evaluation, the Krachmer modified scale, which evaluates the distribution and extent of the corneal guttae and the degree of corneal edema on a slit-lamp examination. However, clinical evaluations have been shown to have a wide degree of interobserver variations [2,4,24,25]. Recent studies have described the Scheimpflug and Anterior Segment Optical Coherence Tomography (AS-OCT) criteria for FECD classification based on changes in the pachymetry map and posterior elevation map, such as the thinnest corneal point displacement, isopach irregularity and posterior corneal elevation. Patel et al. demonstrated that these features predict progression more accurately, far outperforming CCT as a prognostic marker [26,27,28].

Even though FECD is a dystrophy of the endothelium and the Descemet membrane, its impact also extends to the other corneal layers, including in early stages of the disease. These changes are correlated with modified visual quality objective metrics, such as increased high-order aberrations and anterior and posterior corneal face changes. Corneas with early-stage FECD exhibit more anterior and posterior high-order aberrations compared to healthy corneas [8,29], affecting visual quality.

Our study aims to provide a comparison of the subclinical and clinical stages of FECD regarding the corneal structure and visual quality. The objective is to assess these parameters in the two stages of the disease and before and after performing a DMEK intervention.

## 2. Materials and Methods

This is a retrospective observational study. We included patients who underwent DMEK for Fuchs endothelial dystrophy between April 2023 and October 2025 from our service at the Clinical Institute of Ophthalmological Emergencies “Prof. Dr. Mircea Olteanu”.

The study protocol consisted of evaluating visual acuity (best-corrected visual acuity, BCVA, measured on the logMAR scale) and corneal structural and optical quality parameters preoperatively (T0) and at the 6-month follow-up visit (T6), using AS-OCT data obtained with an MS-39 Device for Anterior Segment analysis (Costruzione Strumenti Oftalmici, Firenze, Italy). The evaluated parameters were: the central corneal thickness (CCT); the refractive analysis parameters for the central corneal 3 mm—the mean pupillary power (MPP), anterior corneal astigmatism, posterior corneal astigmatism, and total corneal astigmatism; the optical quality parameters–the point spread function (PSF), measured by the Strehl ratio; the anterior, posterior and total corneal high-order aberrations (HOAs) for the 3 mm central corneal zone and the HOAs for the 6 mm central corneal zone, calculated by the root mean square (RMS) of the HOAs using the Zernike terms up to the seventh order. We also evaluated the Gullstrand ratios and the anterior and posterior corneal regularity indices (anterior RMS/A, posterior RMS/A).

The inclusion criteria consisted of patients diagnosed with Fuchs endothelial dystrophy. The exclusion criteria were: significant corneal fibrosis and ocular surface disease, other corneal diseases, vitreoretinal pathology, previous ocular inflammation, history of multiple corneal transplantation surgeries, vitrectomy, aphakia, ocular trauma, persistent graft detachment, primary graft failure, patients lost during follow-up, and systemic diseases (diabetes, autoimmune disorders, infectious diseases). Following the inclusion and exclusion criteria, out of the total 71 patients who underwent DMEK for FECD in the April 2023–October 2025 interval, only 40 patients (48 eyes) were included in the study. Statistical analyses were performed at the eye level, with each operated eye considered as the unit of analysis. Both eyes from the same patient were included when they independently met the inclusion criteria and underwent DMEK. This approach was chosen because the study focused on corneal remodeling according to disease grading severity and surgical indication, which were assessed separately for each eye. We divided the patients into 2 subgroups following the criteria described by the new AS-OCT severity grading [26,28]: a subclinical edema group (F-SCE), with subclinical edema and with pachymetry map changes (displacement of corneal thinnest point, loss of isopach regularity, posterior elevation on the central 3 mm of the posterior elevation map); and a clinical edema (F-CE) group, with a clinically evident edema together with pachymetry map alterations.

To our knowledge, this is the first study assessing the corneal evolution of patients with FECD who underwent DMEK in a Romanian cohort.

All surgeries were done using the same surgical technique and were performed by the same experienced surgeon (A.G.G.). All the patients in the study followed the same perioperative protocol. After DMEK, all patients underwent postoperative topical treatment with a fixed combination of netilmicin 5 mg/mL and topical dexamethasone 1 mg/mL drops q6d, topical sodium chloride 5% solution q4d, and artificial tears q6d for the first month postoperatively, after which the treatment was tapered.

This study was conducted in accordance with the Declaration of Helsinki and its subsequent amendments. Ethical approval was obtained from the Ethics Committee of the Clinical Institute of Ophthalmological Emergencies “Prof. Dr. Mircea Olteanu” (Nr. 1256/March 2026). Written informed consent was obtained from all patients involved in the study.

The statistical analysis was done using jamovi v. 2.6.44.0 (the jamovi project (2024), jamovi (Version 2.6) [computer software], retrieved from https://www.jamovi.org). The continuous variables were expressed as the mean ± SD, and the categorical variables as counts and percentages. Postoperative change was defined as the 6-month value minus the preoperative value. Normality of variables and change scores were assessed using a Shapiro–Wilk test. For the paired preoperative versus 6-month comparisons of the same eyes, a paired t-test was used when the change scores were approximately normally distributed, and a Wilcoxon signed-rank test was applied otherwise. For the comparisons between independent groups, including Fuchs stage 2 versus stage 3 or postoperative stage-based comparisons, a Welch t-test was used for the approximately normally distributed data because of its robustness to unequal variances and unequal group sizes; otherwise, a Mann–Whitney U test was used. All subgroup analyses should be interpreted as exploratory.

## 3. Results

The study included 48 eyes, of which 36 (75.0%) were from female patients and 12 (25.0%) from male patients. The mean age was 71.3 ± 6.5 years (median 71 years, range 55–86 years) (Table 1).

### 3.1. Overall Cohort Changes: Preoperative to Six-Month Postoperative Follow-Up

In the overall cohort, visual acuity improved in a statistically significant manner: the logMAR BCVA showed a difference from T6 to T0 of −0.638 (*p* < 0.001). Among the followed corneal parameters, statistically significant improvements were detected in the CCT (Δ = −125.0 µm, *p* < 0.001) and the thinnest corneal point decreased markedly (Δ = −92.1 µm, *p* < 0.001). Significant overall improvement was also observed for the MPP (Δ = −2.353, *p* < 0.001), total HOAs at 3 mm (Δ = −0.334, *p* < 0.001), anterior corneal HOAs (Δ = −0.308, *p* = 0.006), posterior corneal HOAs (Δ = −0.113, *p* < 0.001), anterior corneal regularity (Δ = −0.097, *p* = 0.001), posterior corneal regularity (Δ = −1.000, *p* < 0.001), Gullstrand A/P ratio (Δ = +0.144, *p* < 0.001), Gullstrand P/A ratio (Δ = −0.143, *p* < 0.001), and ACA (Δ = +0.912, *p* = 0.034). By contrast, the Cyl at 3 mm, PCA, HOAs 6 mm, and PSF did not show any significant overall change (Table 1).

### 3.2. Subgroup Analysis of F-SCE and F-CE Changes: Preoperative to Six-Month Follow-Up

When stratified by subgroups, the eyes in the F-CE group showed a more statistically significant postoperative response than the eyes in the F-SCE group—see Table 2.

In the F-SCE group, statistically significant improvements were observed in the BCVA logMAR (Δ = −0.567 ± 0.314, *p* < 0.001), central corneal thickness (Δ = −84.6 ± 48.1 µm, *p* < 0.001), thinnest corneal point (Δ = −74.4 ± 41.0 µm, *p* < 0.001), and MPP (Δ = −1.010 ± 2.183, *p* < 0.001). In addition, the Gullstrand ratios changes were statistically significant, with the A/P increasing (Δ = +0.111 ± 0.089, *p* < 0.001) and the P/A decreasing (Δ = −0.078 ± 0.064, *p* < 0.001), and the posterior corneal regularity improved (Δ = −0.117 ± 0.229, *p* = 0.004). However, no significant change was detected in the anterior corneal surface regularity or optical quality parameters—the total corneal HOAs (3 mm), total corneal HOAs (6 mm), anterior corneal HOAs, posterior corneal HOAs, and PSF. The corneal astigmatism metrics—Cyl, anterior corneal astigmatism, and posterior corneal astigmatism—also showed no statistically significant changes.

**Figure 1:**
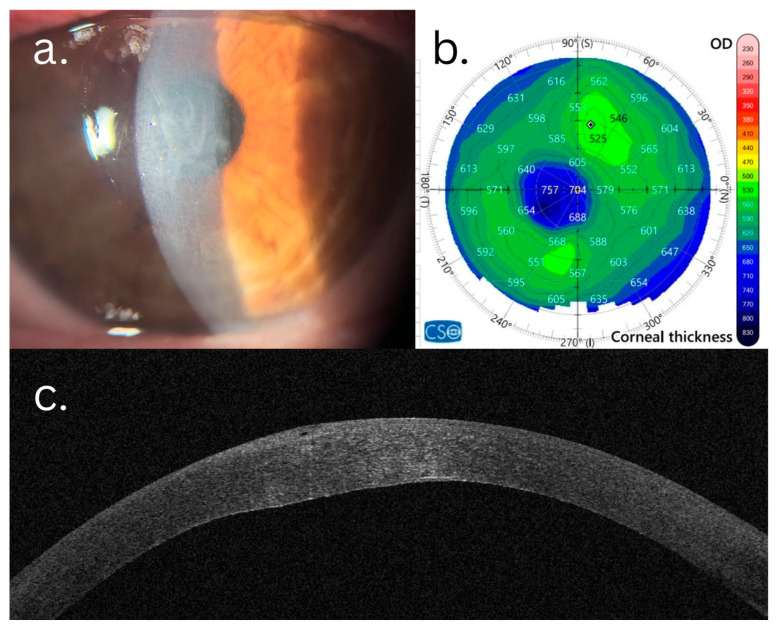

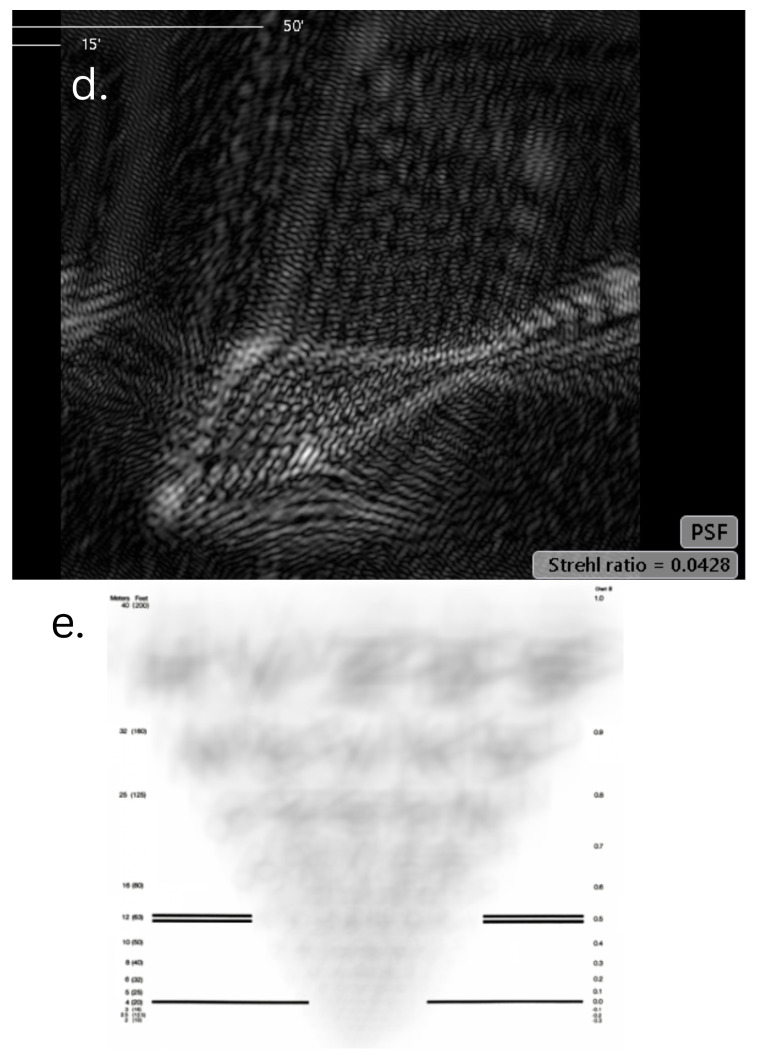
Pre-DMEK aspects of FECD patient with clinical edema: (**a**) clinically evident corneal edema with important stromal haze; (**b**) pachymetry map showing important central corneal edema with marked isopach irregularity; (**c**) AS-OCT slice revealing epithelial dysfunction, stromal hyperreflectivity and posterior corneal irregularity; and (**d**,**e**) point spread function (PSF) analysis. PSF demonstrates diffuse, irregular, and asymmetric distribution of light with increased scatter, reflecting poor optical quality and diminished image sharpness.

**Figure 2:**
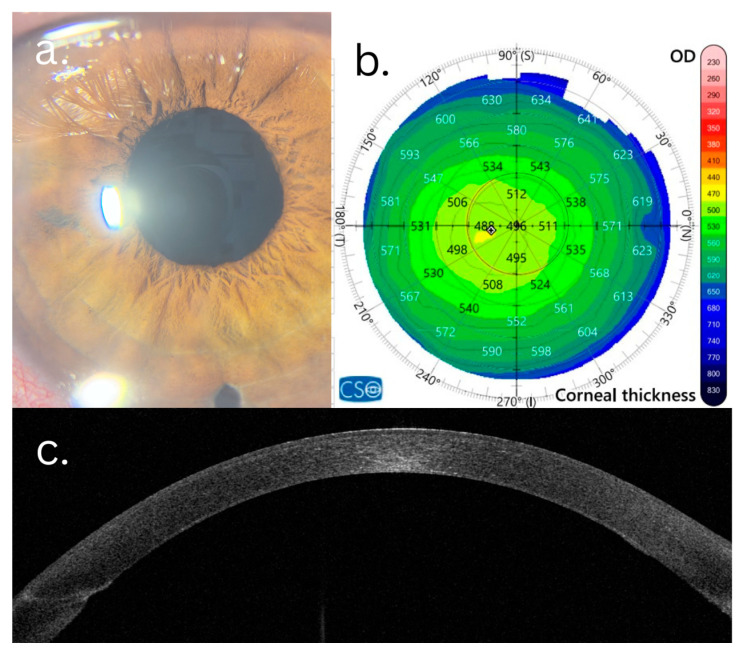

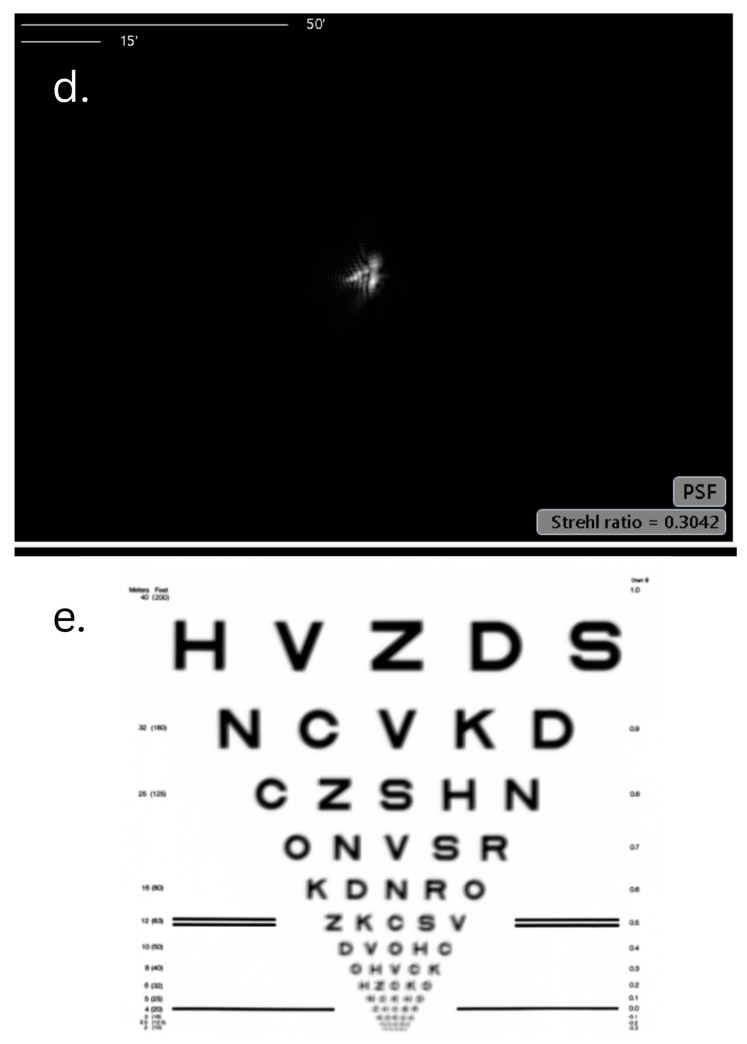
Six-month post-DMEK aspects of FECD patient with clinical edema: (**a**) clear cornea with well-integrated DMEK graft; (**b**) pachymetry map showing normalization of pachymetry values with regularization of isopachs; (**c**) AS-OCT slice revealing well-integrated DMEK graft, with normalization of stromal reflectivity; and (**d**,**e**) PSF demonstrating substantially more compact central intensity peak and marked reduction in light scatter.

In the F-CE group, the magnitude of postoperative changes was greater. Statistically significant improvement was observed in the BCVA logMAR (Δ = −0.729 ± 0.392, *p* < 0.001), with normalization of central corneal thickness (Δ = −176.8 ± 70.1 µm, *p* < 0.001) and of the thinnest corneal point (Δ = −114.8 ± 62.3 µm, *p* < 0.001). In contrast to the F-SCE group, the F-CE eyes also demonstrated significant changes in the anterior corneal astigmatism (Δ = +2.259 ± 3.048, *p* = 0.002), cylinder diopter (Δ = +2.762 ± 3.993, *p* < 0.001), posterior corneal astigmatism (Δ = −1.052 ± 1.829, *p* = 0.004), total corneal HOAs 3 mm (Δ = −0.784 ± 1.202, *p* < 0.001), total corneal HOAs 6 mm (Δ = −1.077 ± 1.838, *p* = 0.005), anterior corneal HOAs (Δ = −0.750 ± 1.239, *p* = 0.002), posterior corneal HOAs (Δ = −0.227 ± 0.169, *p* < 0.001), and PSF (Δ = +0.068 ± 0.192, *p* = 0.008). Both the anterior corneal surface regularity (Δ = −0.231 ± 0.353, *p* = 0.003) and posterior surface regularity (Δ = −2.134 ± 4.684, *p* < 0.001) improved significantly. The Gullstrand A/P ratio increased significantly (Δ = +0.186 ± 0.252, *p* = 0.003), while the Gullstrand P/A ratio decreased significantly (Δ = −0.226 ± 0.333, *p* = 0.003) (Figure 1 and Figure 2).

Comparison of the postoperative changes between the F-SCE and the F-CE groups confirm that the F-CE eyes improved significantly more than the F-SCE eyes for most structural and optical outcomes—see Table 3.

The between-stage difference was significant for the central corneal thickness (*p* < 0.001), thinnest corneal point (*p* = 0.015), anterior corneal astigmatism (*p* = 0.001), cylinder (*p* < 0.001), posterior corneal astigmatism (*p* = 0.003), total corneal HOAs 3 mm (*p* < 0.001), total corneal HOAs 6 mm (*p* = 0.002), anterior corneal HOAs (*p* < 0.001), posterior corneal HOAs (*p* < 0.001), PSF (*p* = 0.003), anterior corneal surface regularity (*p* = 0.002), and posterior corneal surface regularity (*p* < 0.001). By contrast, the between-stage difference in the MPP showed only borderline significance (*p* = 0.051), and the magnitude of changes in the Gullstrand A/P and P/A ratios did not differ significantly between stages (*p* = 0.203 and *p* = 0.208, respectively).

**Figure 3:**
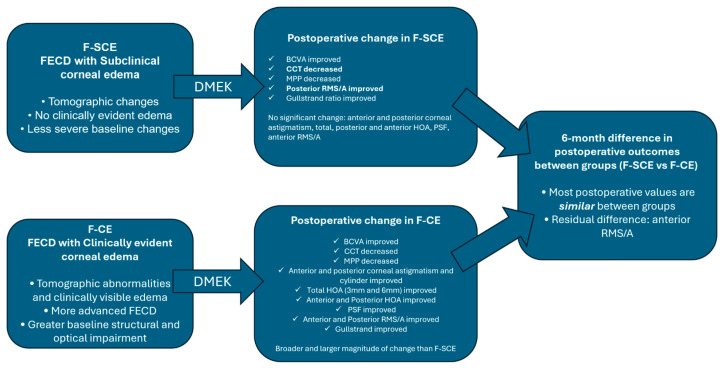
Optical quality improvement in the F-SCE and F-CE groups.

The six-month follow-up outcomes comparison between the F-SCE and the F-CE groups showed that the only statistically significantly different postoperative marker was the anterior corneal regularity (anterior RMS/A, *p* = 0.032) (Table 2, “Postop F-SCE versus F-CE”, Figure 3).

## 4. Discussion

Our study highlights not just that DMEK was associated with marked anatomical and optical recovery by 6 months in eyes with Fuchs endothelial corneal dystrophy, but that the magnitude of this recovery depended strongly on the preoperative disease stage. Although both the eyes with subclinical edema and eyes with clinical edema improved after surgery, the clinical edema eyes showed greater changes across the pachymetric, tomographic, and optical quality parameters. At the same time, most of the 6-month postoperative values were no longer significantly different between the subclinical edema and clinical edema eyes, suggesting that DMEK can substantially normalize the corneal state of more advanced FECD and bring them close to the postoperative profile achieved in less advanced disease.

To our knowledge, this is the first study assessing the corneal evolution in patients with FECD who underwent DMEK in a Romanian cohort. Furthermore, this is one of the few studies to employ the MS-39 platform for a comprehensive corneal optical quality assessment, including the corneal-specific PSF measured by the Strehl ratio, in FECD patients who underwent a DMEK. Previous studies have used Scheimpflug-based devices [30,31] and others [32,33,34] for corneal aberrations and densitometry. The MS-39 derives the Strehl ratio from the corneal wavefront aberration data only [35], allowing for an evaluation of how much the cornea alone degrades image quality.

The evaluation of the overall cohort at six-months post-DMEK surgery showed multiple statistically significant changes, with an important CCT decrease, consistent with previously published data [36,37]. The subgroup analysis showed a significant CCT decrease both in the F-SCE and F-CE groups.

The Gullstrand A/P ratio normalized in both subgroups, reflecting restoration of the anterior-to-posterior curvature relationship as the posterior surface steepens during stromal deturgescence, consistent with the hyperopic shift documented by Ham et al. [38]. Notably, the magnitude of Gullstrand ratio change did not differ between subgroups.

Statistically significant improvements were also present in the BCVA, MPP, and ACA, but, interestingly, not in the PCA. The PCA findings deserve particular attention, as the literature on this parameter after DMEK shows considerable heterogeneity. Significant PCA changes were reported by Agha et al. [39], Alnawaiseh et al. [40] and Chamberlain et al. [41]. Conversely, Guerra et al. [42] and Shajari et al. [43] found no significant change, with the latter concluding that astigmatism change after DMEK is inherently unpredictable. Analyzing by subgroup, we found a divergent pattern; in the F-CE group, both the ACA (Δ = +2.259 D, *p* = 0.002) and PCA (Δ = −1.052 D, *p* = 0.004) changed significantly, whereas in the F-SCE group neither reached significance, suggesting that posterior curvature restoration is proportional to the baseline corneal dysfunction.

The significant ACA change in our F-CE group contrasts with several reports that found the anterior astigmatism stable after DMEK [39,40,44]. However, the anterior corneal surface in advanced FECD undergoes epithelial changes, mainly thickening [45], subepithelial fibrosis [46], and anterior stromal haze [47]. The resolution of these phenomena following DMEK may explain why our F-CE group showed significant ACA changes not observed in milder cohorts.

Interestingly, the PSF did not show a statistically significant decrease in the overall cohort. Although the PSF did not change significantly in the overall cohort, it improved significantly in the F-CE group but not in the F-SCE group, and the between-stage difference in the change was significant. This is an important example of how pooled analyses can mask severity-dependent biological effects. Optical quality metrics may not behave uniformly across all eyes with FECD, and the absence of a significant effect in the whole cohort should not necessarily be interpreted as absence of meaningful postoperative optical change. In this study, the stage-based analysis revealed that several parameters with modest or non-significant overall effects actually changed substantially in more advanced disease. A notable example is the MPP, which showed only borderline statistical significance (*p* = 0.051), but the numerical difference was notable, with a larger decrease in the F-CE eyes than in the F-SCE eyes. Given the modest sample size, this borderline result should not be interpreted as evidence of no effect. Rather, it suggests that the MPP may reflect part of the refractive and corneal power remodeling that accompanies postoperative deturgescence, particularly in clinically edematous eyes. A larger cohort would be needed to determine whether this parameter has independent clinical relevance.

The anterior, posterior, and total HOAs at 3 mm showed significant changes in the overall cohort. The total corneal HOAs improvement after DMEK in FECD patients is consistent with previously published studies [48]. Regarding the anterior and posterior HOAs, they follow different post-DMEK improvement paths. Rudolph et al. and Duggan et al. described a decrease in posterior HOAs after DMEK [30,49]. Regarding anterior HOAs, Van Dijk et al. and Duggan et al. reported no significant anterior HOAs difference post-DMEK [49,50]. Hayashi et al. showed that anterior HOAs decreased only gradually, improving from the 6-month follow-up to the 12-month follow-up, requiring a longer time to stabilization than posterior HOAs. The total HOAs improved, but remained significantly above those of healthy controls [48].

These findings also contribute to the clinical discussion regarding the optimal timing of DMEK in FECD. In the present cohort, the eyes with clinically evident edema achieved 6-month postoperative outcomes largely comparable to those of eyes with subclinical edema. The data suggest that, in the absence of significant corneal fibrosis, DMEK can still produce substantial structural and optical recovery even when surgery is performed at the clinically edematous stage. Therefore, the critical threshold for surgical timing may not be the presence of edema alone, but rather the development of irreversible stromal remodeling. However, this postoperative convergence should be interpreted as convergence between the FECD subgroups, not as complete normalization to healthy cornea values, since previous studies have shown that post-DMEK eyes may retain residual differences in corneal HOAs, posterior corneal curvature, pachymetric distribution, and optical quality compared with normal corneas [48,51].

The subgroup analysis revealed that the total, anterior and posterior HOAs improved significantly only in the F-CE group, but not in the F-SCE group. This, together with the fact that the statistically significant changes in the F-SCE subgroup were in the CCT and posterior corneal regularity (posterior RMS/A), could point towards the possible importance of posterior corneal regularity metrics (posterior RMS/A) as a complementary marker that should be taken into consideration when considering DMEK in a subclinical edema patient. The posterior RMS/A should be considered an exploratory marker, complementary to the established tomographic indicators of subclinical edema in FECD, which include loss of regular or parallel isopachs, displacement of the thinnest point, and focal posterior corneal surface depression [26,27,28].

Taking into account that FECD is a relatively rare disease, this relatively low number of patients represent a promising future direction. A limitation is that the furthest postoperative follow-up included in this study was at 6 months. Published longitudinal DMEK studies suggest that the 6-month visit captures the major early phase of postoperative corneal changes, but not necessarily the full extent of optical and tomographic remodeling. Serial AS-OCT and Scheimpflug studies have shown that central pachymetry decreases rapidly, mostly within the first 1–6 months, whereas HOAs reduction, corneal densitometry, and visual acuity may continue to improve for 12 to 24 months [36,50,52,53]. We plan to extend the research by studying the corneal changes at later follow-up points, such as the one-year and two-years marks. We also acknowledge the lack of endothelial specular microscopy data in the statistical analysis.

The present study should consequently not be interpreted as an assessment of endothelial graft survival or endothelial reserve, but rather as an evaluation of AS-OCT-derived corneal remodeling and optical quality changes after DMEK. Similar endpoint-specific approaches have been used in previous FECD studies that focused on visual quality, contrast sensitivity, quality of life, epithelial thickness, or corneal morphology rather than postoperative endothelial cell metrics after DMEK or DSAEK. This is due to the fact that the specular microscopy data were acquired using multiple devices, making them unreliable for statistical analysis.

In advanced-stage FECD, posterior corneal ripples have been shown to be a promising biomarker for visual recovery and for postoperative evolution [54]. We propose that the posterior corneal regularity (posterior RMS/A) metrics might also be especially useful in early-stage FECD patients, as they could represent an early marker of corneal dysfunction.

## 5. Conclusions

Overall, these findings indicate that despite worse baseline disease and greater postoperative changes in the clinical edema eyes, the 6-month postoperative values had largely converged to levels similar to those observed in the subclinical edema eyes. This suggests that early surgery may interrupt the cycle of edema-related remodeling, while also reassuring patients with clinically evident edema that they can still experience marked anatomical and optical rehabilitation after DMEK. Further research is needed to evaluate the predictive value of posterior corneal irregularity as a marker of corneal dysfunction in FECD eyes with subclinical edema.

## Figures and Tables

**Table 1:** Comparison of preoperative and postoperative corneal changes in the entire cohort, with significance of before–after differences. * Represents statistically significant changes.

Parameter	Overall T0 (Mean ± SD)	Overall T6 (Mean ± SD)	Overall Δ (Mean ± SD)	Significance Test (*p* Value)
CCT (µm) *	644.5 ± 69.5	519.5 ± 59.8	−125.0 ± 74.2	paired t (<0.001)
Thinnest corneal point (µm) *	597.8 ± 58.0	505.8 ± 58.1	−92.1 ± 54.7	paired t (<0.001)
ACA 3 mm (D) *	−2.390 ± 2.400	−1.478 ± 1.259	+0.912 ± 2.443	Wilcoxon (0.034)
MPP 3 mm (D) *	45.3 ± 4.2	43.0 ± 2.4	−2.353 ± 4.178	Wilcoxon (<0.001)
Cylinder 3 mm (D)	−2.712 ± 3.015	−1.670 ± 1.075	+1.042 ± 3.162	Wilcoxon (0.102)
PCA 3 mm (D)	0.958 ± 1.289	0.544 ± 0.343	−0.413 ± 1.342	Wilcoxon (0.138)
Total corneal HOAs 3 mm (µm) *	0.649 ± 0.851	0.315 ± 0.317	−0.334 ± 0.924	Wilcoxon (<0.001)
Total corneal HOAs 6 mm (µm)	1.568 ± 1.425	1.191 ± 0.777	−0.377 ± 1.534	Wilcoxon (0.169)
Anterior corneal HOAs (µm) *	0.622 ± 0.873	0.314 ± 0.321	−0.308 ± 0.939	Wilcoxon (0.006)
Posterior corneal HOAs (µm) *	0.189 ± 0.152	0.076 ± 0.055	−0.113 ± 0.163	Wilcoxon (<0.001)
PSF (Strehl ratio)	0.210 ± 0.147	0.222 ± 0.094	+0.012 ± 0.170	Wilcoxon (0.442)
Anterior corneal surface regularity (Anterior RMS/A) (µm/mm) *	0.200 ± 0.254	0.103 ± 0.105	−0.097 ± 0.275	Wilcoxon (0.001)
Posterior corneal regularity (Posterior RMS/A) (µm/mm) *	1.223 ± 3.214	0.224 ± 0.164	−1.000 ± 3.223	Wilcoxon (<0.001)
Gullstrand A/P ratio *	1.091 ± 0.187	1.235 ± 0.044	+0.144 ± 0.181	Wilcoxon (<0.001)
Gullstrand P/A ratio *	0.954 ± 0.239	0.811 ± 0.029	−0.143 ± 0.234	Wilcoxon (<0.001)

Abbreviations: ACA = anterior corneal astigmatism; CCT = central corneal thickness; D = diopters; MPP = mean pupillary power; Cyl = total corneal cylinder value at 3 mm; HOAs = high-order aberrations; PCA = posterior corneal astigmatism; PSF = point spread function.

**Table 2:** Comparison of preoperative and postoperative corneal changes in the subclinical corneal edema subgroup (F-SCE) and the clinically evident corneal edema subgroup (F-CE), with significance of before–after differences.

Parameter	F-SCE T0 (Mean ± SD)	F-SCE T6 (Mean ± SD)	F-SCE Test (*p* Value)	F-CE T0 (Mean ± SD)	F-CE T6 (Mean ± SD)	F-CE Test (*p* Value)	Postop F-SCE Versus F-CE (*p* Value)
CCT (µm)	603.9 ± 48.5	519.2 ± 64.0	Wilcoxon (<0.001)	696.7 ± 56.8	519.9 ± 55.5	Wilcoxon (<0.001)	Mann–Whitney U (0.893)
Thinnest corneal point (µm)	578.9 ± 45.9	504.5 ± 63.7	paired t (<0.001)	622.1 ± 63.7	507.4 ± 51.5	paired t (<0.001)	Mann–Whitney U (0.983)
ACAm (D) 3 mm	−1.430 ± 1.271	−1.516 ± 1.368	paired t (0.703)	−3.686 ± 2.941	−1.428 ± 1.126	Wilcoxon (0.002)	Mann–Whitney U (1.000)
MPP (D) 3 mm	44.12 ± 2.17	43.11 ± 2.45	Wilcoxon (<0.001)	46.90 ± 5.55	42.82 ± 2.29	paired t (0.002)	Welch t (0.670)
Cylinder (D) 3 mm	−1.512 ± 1.326	−1.808 ± 1.173	Wilcoxon (0.077)	−4.256 ± 3.826	−1.494 ± 0.932	Wilcoxon (<0.001)	Mann–Whitney U (0.394)
PCA (D) 3 mm	0.532 ± 0.307	0.592 ± 0.303	paired t (0.486)	1.533 ± 1.814	0.480 ± 0.389	Wilcoxon (0.004)	Mann–Whitney U (0.568)
Total corneal HOAs 3 mm (µm)	0.292 ± 0.180	0.308 ± 0.352	Wilcoxon (0.107)	1.108 ± 1.125	0.324 ± 0.274	Wilcoxon (<0.001)	Mann–Whitney U (0.339)
Total corneal HOAs 6 mm (µm)	1.031 ± 0.663	1.199 ± 0.872	Wilcoxon (0.216)	2.259 ± 1.820	1.181 ± 0.656	Wilcoxon (0.005)	Mann–Whitney U (0.685)
Anterior corneal HOAs (µm)	0.269 ± 0.185	0.305 ± 0.362	Wilcoxon (0.876)	1.077 ± 1.167	0.327 ± 0.268	Wilcoxon (0.002)	Mann–Whitney U (0.200)
Posterior corneal HOAs (µm)	0.106 ± 0.064	0.082 ± 0.067	Wilcoxon (0.074)	0.295 ± 0.166	0.068 ± 0.035	paired t (<0.001)	Mann–Whitney U (0.706)
PSF (Strehl ratio)	0.250 ± 0.118	0.217 ± 0.097	paired t (0.240)	0.159 ± 0.167	0.227 ± 0.093	Wilcoxon (0.008)	Welch t (0.729)
Anterior corneal regularity (Anterior RMS/A) (µm/mm)	0.090 ± 0.064	0.097 ± 0.124	Wilcoxon (0.124)	0.342 ± 0.330	0.111 ± 0.077	Wilcoxon (0.003)	Mann–Whitney U (0.032)
Posterior corneal regularity (Posterior RMS/A) (µm/mm)	0.329 ± 0.197	0.211 ± 0.125	Wilcoxon (0.004)	2.374 ± 4.664	0.240 ± 0.205	Wilcoxon (<0.001)	Mann–Whitney U (0.739)
Gullstrand A/P ratio	1.125 ± 0.070	1.236 ± 0.045	paired t (<0.001)	1.048 ± 0.269	1.234 ± 0.042	paired t (0.003)	Welch t (0.892)
Gullstrand P/A ratio	0.889 ± 0.053	0.811 ± 0.030	paired t (<0.001)	1.038 ± 0.343	0.812 ± 0.029	Wilcoxon (0.003)	Welch t (0.926)

**Table 3:** Comparison of postoperative—preoperative corneal changes in the subclinical corneal edema subgroup (F-SCE) and the clinically evident corneal edema subgroup (F-CE), with significance of between-group differences.

Parameter	F-SCE Δ (Mean ± SD)	F-CE Δ (Mean ± SD)	F-CE Test (*p* Value)	Between-Stage Test on Δ (*p* Value)
CCT (µm)	−84.6 ± 48.1	−176.8 ± 70.1	Wilcoxon (<0.001)	Mann–Whitney U (<0.001)
Thinnest corneal point (µm)	−74.4 ± 41.0	−114.8 ± 62.3	paired t (<0.001)	Welch t (0.015)
ACA (D) 3 mm	−0.086 ± 1.157	+2.259 ± 3.048	Wilcoxon (0.002)	Mann–Whitney U (0.001)
MPP (D) 3 mm	−1.010 ± 2.183	−4.080 ± 5.409	paired t (0.002)	Mann–Whitney U (0.051)
Cylinder (D) 3 mm	−0.296 ± 1.248	+2.762 ± 3.993	Wilcoxon (<0.001)	Mann–Whitney U (<0.001)
PCA (D) 3 mm	+0.060 ± 0.441	−1.052 ± 1.829	Wilcoxon (0.004)	Mann–Whitney U (0.003)
Total corneal HOAs 3 mm (µm)	+0.016 ± 0.377	−0.784 ± 1.202	Wilcoxon (<0.001)	Mann–Whitney U (<0.001)
Total corneal HOAs 6 mm (µm)	+0.167 ± 0.975	−1.077 ± 1.838	Wilcoxon (0.005)	Mann–Whitney U (0.002)
Anterior corneal HOAs (µm)	+0.036 ± 0.367	−0.750 ± 1.239	Wilcoxon (0.002)	Mann–Whitney U (<0.001)
Posterior corneal HOAs (µm)	−0.024 ± 0.085	−0.227 ± 0.169	paired t (<0.001)	Mann–Whitney U (<0.001)
PSF (Strehl ratio)	−0.032 ± 0.139	+0.068 ± 0.192	Wilcoxon (0.008)	Mann–Whitney U (0.003)
Anterior corneal surface regularity (Anterior RMS/A) (µm/mm)	+0.007 ± 0.122	−0.231 ± 0.353	Wilcoxon (0.003)	Mann–Whitney U (0.002)
Posterior corneal regularity (Posterior RMS/A) (µm/mm)	−0.117 ± 0.229	−2.134 ± 4.684	Wilcoxon (<0.001)	Mann–Whitney U (<0.001)
Gullstrand A/P ratio	+0.111 ± 0.089	+0.186 ± 0.252	paired t (0.003)	Welch t (0.203)
Gullstrand P/A ratio	−0.078 ± 0.064	−0.226 ± 0.333	Wilcoxon (0.003)	Mann–Whitney U (0.208)

## Data Availability

The original contributions presented in this study are included in the article. Further inquiries can be directed to the corresponding author.

## Abbreviations

The following abbreviations are used in this manuscript:

ACA	anterior corneal astigmatism
AS-OCT	anterior segment optical coherence tomography
BCVA	best-corrected visual acuity
CCT	central corneal thickness
Cyl	total corneal cylinder value at 3 mm
D	diopters
DMEK	Descemet membrane endothelial keratoplasty
DSAEK	Descemet stripping endothelial keratoplasty
FECD	Fuchs endothelial corneal dystrophy
F-CE	Fuchs dystrophy with clinically evident corneal edema
F-SCE	Fuchs dystrophy with subclinical corneal edema
HOAs	high-order aberrations
RMS	root mean square
MPP	mean pupillary power
PCA	posterior corneal astigmatism
PSF	point spread function
